# Partial Removal of Phenolics Coupled with Alkaline pH Shift Improves Canola Protein Interfacial Properties and Emulsion in In Vitro Digestibility

**DOI:** 10.3390/foods10061283

**Published:** 2021-06-04

**Authors:** Jiang Jiang, Yunqing Nie, Xuemei Sun, Youling L. Xiong

**Affiliations:** 1 School of Food Science and Technology, Jiangnan University, Wuxi 214122, China; jiangjiang@jiangnan.edu.cn (J.J.); nieyq219@gmail.com (Y.N.); sunxm0701@163.com (X.S.); 2 Department of Animal and Food Sciences, University of Kentucky, Lexington, KY 40546, USA

**Keywords:** canola protein isolate, dephenol, pH_12_ shift, solubility, emulsifying properties, in vitro digestion

## Abstract

The effect of polyphenol removal (“dephenol”) combined with an alkaline pH shift treatment on the O/W interfacial and emulsifying properties of canola seed protein isolate (CPI) was investigated. Canola seed flour was subjected to solvent extraction to remove phenolic compounds, from which prepared CPI was exposed to a pH_12_ shift to modify the protein structure. Dephenoled CPI had a light color when compared with an intense dark color for the control CPI. Up to 53% of phenolics were removed from the CPI after the extraction with 70% ethanol. Dephenoled CPI showed a partially unfolded structure and increased surface hydrophobicity and solubility. The particle size increased slightly, indicating that soluble protein aggregates formed after the phenol removal. The pH_12_ shift induced further unfolding and decreased protein particle size. Dephenoled CPI had a reduced β subunit content but an enrichment of disulfide-linked oligopeptides. Dephenol improved the interfacial rheology and emulsifying properties of CPI. Although phenol removal did not promote peptic digestion and lipolysis, it facilitated tryptic disruption of the emulsion particles due to enhanced proteolysis. In summary, dephenol accentuated the effect of the pH shift to improve the overall emulsifying properties of CPI and emulsion in in vitro digestion.

## 1. Introduction

Canola, the second-largest oilseed crop after soybeans, is valued for its oil quality and fatty acid profile suitable for the cardiovascular system of humans [1]. After the oil extraction, canola protein, as a major by-product, is generally utilized as animal feed. However, canola protein has a well-balanced amino acid composition, the sulfur amino acid content is relatively high (3–4 g/100 g), and its protein efficiency ratio (2.64) exceeds that of soy protein (2.19) [2]. Hence, canola protein has the potential for nutritional as well as techno-functional (e.g., gelation and emulsification) food ingredient development [3].

In the mature seeds of canola exist two types of storage proteins, cruciferin globulin (11 S or 12 S) and napin albumin (2 S or 1.7 S). They are stored in vacuoles of the cotyledon cell and account for, respectively, 60% and 20% of the total protein. In addition, small amounts of oil body protein, trypsin inhibitors, and lipid transfer protein have been reported [4]. Cruciferin is a 300–500 kDa hexamer composed of six subunits, 3 *α*-chains and 3 *β*-chains, which are linked by inter-chain disulfide bonds [5]. Napin consists of two polypeptide chains, a heavy chain (H, 9 kDa) and a light chain (L, 4 kDa), which are also linked by two inter-chain disulfide bonds [6].

The application of canola protein in the food industry is currently limited due to its poor solubility, which is attributed, in part, to the presence of polyphenols, phytic acid, and glucosinolates that bind to proteins. In addition, these components have adverse effects on the digestibility and sensory quality of the protein, including dark color, bitter taste, and astringency [7]. Phenolic compounds present in canola are mostly phenolic acids (e.g., sinapic acid and sinapine) and tannins. Both free and bound forms of phenolic acids as well as their esters and glycosides have been isolated [8]. The total phenolic content in canola seeds, on a dry weight basis, is almost 30 times that of soybean [9].

Phenolic compounds can interact with protein through different mechanisms, including reversible hydrophobic association, electrostatic attraction, hydrogen bonding, and irreversible covalent adduction [10]. Under oxidative conditions, the phenol moiety can be converted to quinones, which in turn, modify protein functional groups. The binding of polyphenols to sulfhydryl and amino groups reduces protein digestibility and bioavailability and hampers protein solubility [10]. Therefore, it is desirable to remove phenolic substances from canola meal so as to augment the functionality and nutritional quality of its protein.

Apart from dephenol, the structural modification of proteins through physicochemical means can have a positive effect on protein functionality. Chemical modifications include succinylation [11], acetylation, and maleylation [12], while enzymatic modifications include limited hydrolysis and, conversely, cross-linking [13]. pH_12_ shift processing, an emerging technique to modify protein structures, has been successfully applied to improve the functionality of legume proteins [14,15]. By exposure to an extreme pH condition followed by neutralization, this treatment induces a molten globule conformation in soy protein, enabling strong surface activity and emulsifying capacity [14].

The objective of this research was to investigate the effect of polyphenol removal from canola meal on the structural and emulsifying properties of canola protein isolate (CPI) and the emulsion digestibility. Dephenoled CPI was subjected to a pH_12_ shift to determine whether additional improvement in functional properties could be achieved.

## 2. Materials and Methods

### 2.1. Materials

Dehulled canola seeds (*Brassica napus* L.) were donated by the Oil Crop Research Institute of the Chinese Academy of Agricultural Sciences (Beijing, China). Corn oil (Luhua, Laiyang, China) was stripped by means of Florisil to eliminate phospholipids and any polar compounds. All chemical reagents (MilliporeSigma, St. Louis, MO, USA) were of analytical grade.

### 2.2. Removal of Phenolic Compounds

The dehulled and milled canola seed flour was treated with n-hexane to extract the oil, and the extraction process was repeated three times [16]. Phenolic compounds in the defatted canola flour were removed using the method of Vuorela, Meyer, and Heinonen [17]. The phenolic compounds were extracted at room temperature with an ethanol solvent (ethanol-to-water ratio of 7:3 (*v*/*v*)) at a flour-to-solvent ratio of 1:10 (*w*/*v*). The dephenoled flour was placed in a fume hood overnight to evaporate the solvents.

### 2.3. Preparation of Protein Isolate and pH_12_ Shift Treatment

The CPI was prepared from the dephenoled flour or defatted-only flour (as a control) according to the procedure of Pirestani et al. [18]. The flours were dispersed in deionized water (1:10 (*w*/*v*)), adjusted to pH 11 with 2 M NaOH, and then stirred for 2 h at room temperature (21 ± 1 °C). The dispersions were centrifuged at 8000× *g* for 30 min, and the collected supernatants were adjusted to pH 4.5 with 1 M HCl, followed by centrifugation at 5000× *g* for 20 min. The precipitate was washed three times with deionized water and adjusted to pH 7.0, lyophilized, and then stored at −20 °C.

For the pH_12_ shift, the lyophilized CPI was suspended in deionized water to obtain a 20 mg/mL protein concentration. The dispersions were adjusted to pH 12 with 2 M NaOH, held for 1 h at room temperature, then neutralized to pH 7.0 with 2 M HCl, followed by stirring for 1 h [14]. The resulting proteins were expected to possess a molten globule structure.

### 2.4. Determination of Total Phenolic Content (TPC)

The TPC in the defatted canola seed flour and in the CPI samples was determined according to the method by Labuckas et al. [19]. The phenolic compounds were extracted with the 70% ethanol solution (ethanol-to-H_2_O ratio of 70:30 (*v/v*), the same solvent system as used above for the phenol removal). Folin–Ciocalteu’s reagent (2.0 mL) was used to react with the phenolics for 90 min in the dark, and the absorbance was measured at 765 nm. A reference curve was constructed using gallic acid as the standard. The TPC was expressed as mg of gallic acid equivalents per g of the extract.

### 2.5. UPLC-PAD-QTOF-MS Analysis of Extracted Phenolic Compounds

The phenolic compounds were separated using an Acquity UPLC system (Waters, Milford, MA, USA) equipped with a binary solvent delivery system, an autosampler, and a BEH C18 column (2.1 × 100 mm, 1.7 µm particle size). Detailed conditions were established based on a previous report with some modifications [20]. Briefly, the mobile phase was prepared in a gradient elution mode with acetonitrile (Solvent A) and 0.1% formic acid (Solvent B) as follows: 0–0.1 min, 2% A and 98% B; 15 min, 20% A and 80% B; 20 min, 40% A and 60% B; 25 min, 80% A and 20% B; 27 min, 100% A and 0% B. The total run time was 30 min. The flow rate was 300 µL/min and the injection volume was 1 μL. Detection was done with a multi-reaction monitoring scan mode [21]. The compounds were analyzed in ES^−^ and ES^+^ modes. The positive mode operating conditions were as follows: desolvation gas 700 L/h at 400 °C, cone gas 50 L/h, source temperature of 100 °C, capillary of 3.0 kV, and cone voltage of 30 V. The negative mode conditions were as follows: capillary of 3.5 kV and cone voltage of 20 V. The data were acquired and analyzed by the Waters MassLynx V4.1 software (Waters, Milford, MA, USA).

### 2.6. Protein Solubility

The Biuret method was used to determine the protein solubility, and bovine serum albumin (BSA) was used as the standard [22]. Aliquots of the protein solution (1 mL) were added to 1.5-mL microcentrifuge tubes and centrifuged at 10,000× *g* for 10 min. Protein solubility was defined as the percentage of the supernatant protein concentration over the total protein concentration in the dispersion.

### 2.7. Electrophoresis

SDS–PAGE was performed to elucidate the protein profiles in the control and treated CPI samples using a stacking gel and a resolving gel containing 4% and 12% acrylamide, respectively. The protein sample was prepared with and without 5% *β*-mercaptoethanol. Aliquots of 30 μg protein were loaded onto each lane of the gel. Electrophoresis was run with constant voltages of 30 V while the samples were running through the stacking gel and 100 V when running through the resolving gel.

### 2.8. Dynamic Light Scattering (DLS)

The diluted protein solution (2 mg/mL) was subjected to DLS analysis using a Zetasizer Nano (Malvern Instruments Ltd., Worcestershire, UK). The hydrodynamic diameter of the protein particles was measured, and all measurements were done in triplicate.

### 2.9. Structure Characterization

#### 2.9.1. Surface Hydrophobicity

The surface hydrophobicity was measured using 1-anilino-8-naphthalenesulfonate (ANS; Sigma-Aldrich, St. Louis, MO, USA) as a fluorescence probe [9]. The fluorescence intensity was measured using an excitation wavelength of 365 nm and an emission wavelength of 484 nm in an F7000 fluorescence spectrophotometer (Hitachi, Tokyo, Japan). The initial slope of the linear regression of fluorescence intensity against protein concentration was used as the protein surface hydrophobicity.

#### 2.9.2. Far-UV Circular Dichroism (CD) Spectroscopy

The protein solutions were prepared with deionized water to a 200 μg/mL concentration. The samples were scanned in the range of 190–250 nm using a MOS-450 CD spectrometer (Biologic, Claix, France). There were 5 scans averaged to obtain 1 spectrum. The mean ellipticity was calculated as the following:

[*θ*] (deg cm^2^ dmol^−1^) = (100 × X × M)/ (L × C) (1)

where X is the ellipticity in degree obtained by the CD spectrometer, M is the average molecule weight, C is the protein concentration in mg/mL, and L is the cell path length (cm). The measurements were performed in a quartz cuvette of 1 mm at a scan rate of 100 nm/min.

### 2.10. Interfacial Properties

The dynamic adsorption of the proteins at the oil–water interface was investigated with a DSA100 Analyzer (Krüss, Hamburg, Germany) using the pendant drop method. In the process of measuring the dynamic surface pressure (π), a drop of 8 μL was automatically created by a software-controlled automatic dosing system after a 5 s stabilization. Droplet images were continuously viewed and captured using a video image acquisition system with a CCD camera, and then analyzed according to the Young–Laplace formula. The Ward–Tordai diffusion model was adopted to analyze the diffusion kinetics of proteins at the interface [23]:

(2) $$\pi \left(t\right)=2C_{0}KT{\left(\frac{k_{diff}t}{3.14}\right)}^{\frac{1}{2}}$$

where $C_{0}$ is the concentration of the initial protein solution, K is the Boltzmann constant, T is the absolute temperature, and $k_{diff}$ is the diffusion coefficient.

The surface dilatational properties were measured on a DSA100 Analyzer (Krüss, Hamburg, Germany) using the EDM/ODM module. The amplitude and frequency were maintained at 0.2 and 0.1 Hz, respectively. The surface dilatational modulus, E, was acquired through analyzing the surface area change [24].

### 2.11. Emulsification and Emulsion Properties

The CPI solutions were diluted with deionized water to a concentration of 10 mg/mL. Emulsions were prepared by homogenization of the protein solution with soybean oil (3:1 (*v*/*v*)) at 13,500 rpm for 2 min using an Ultra-Turrax (Ika T18 Basic, Staufen, Germany). The pre-homogenized emulsion was then finely homogenized 3 times with an AH-2010 homogenizer (ATS Engineering Inc., Ontario, Canada) at an overall pressure of 30 MPa.

The freshly prepared emulsions were viewed under an ECLIPSE 80i Nikon microscope (Nikon, Tokyo, Japan). The emulsion samples (10 μL) were transferred to a glass slide with a cover slip and observed with a 100× objective. A Nikon DS-Ri1 camera was used to record the microscopic images.

The emulsion stability was determined using a Turbiscan Lab (Formulaction, Toulouse, France). The data of transmitted light and backscattered light were collected by scanning the sample within a certain period. The stability of the emulsion could be predicted by comparing the changes in the backscattered light intensity of different samples at the same scanning time and then calculated to obtain the Turbiscan Stability Index (TSI) [25]. The emulsions were scanned for 60 min at 30 °C.

(3) $$\mathrm{TSI}=\frac{\sqrt{\sum_{i=1}^{n}{\left(x_{i}-x_{BS}\right)}^{2}}}{n-1}$$

where *n* is the number of scans, *x_i_* is the mean backscattering and transmission for each scan in an experiment, and *x_BS_* is the mean *x_i_*. A high *TSI* value indicates instability and a high probability of phase separation.

### 2.12. In Vitro Emulsion Digestion and Characterization

Each CPI-stabilized emulsion was passed through a simulated stomach and small intestine digestion system [26] with simulated gastric fluid (SGF) containing pepsin (0.32% (*w*/*v*)), NaCl (0.2% (*w*/*v*)), and HCl (0.7%, (*v*/*v*)). A total of 40 g of the initial emulsion was mixed with 40 g of the SGF and adjusted to pH 2.0 with 1.0 M HCl. The mixture was stirred for 1 h at 37 °C. Subsequently, the pH was adjusted to 7.5, and 10 g of the simulated intestine fluid (SIF) (final concentration of 1.6 mg/g lipase, 1.0 mg/g trypsin, and 10 mg/g bile salts) was added to 40 g of the emulsion–SGF mixture. The simulated intestine phase of digestion was incubated at 37 °C for 1 h.

The microstructure of the emulsion digestion products was observed by confocal laser scanning microscopy (Leica Microsystems Inc., Heidelberg, Germany). Diluted samples were stained with fluorescein isothiocyanate dye (FITC) (0.1% (*w*/*v*)) and Nile Red (0.1% (*w*/*v*)). The images were immediately taken when the excitation wavelengths of FITC and Nile Red were 552 nm and 488 nm, respectively.

SDS–PAGE, using the procedure described above, was performed to elucidate the protein degradation pattern. The digested emulsion samples were mixed at a 1:1 (*v/v*) ratio with SDS–PAGE sample buffer and centrifuged at 10,000× *g* for 10 min to collect the subnatant. The stacking and the resolving gels contained 4% and 16% acrylamide, respectively, and electrophoresis was conducted as described above. A total of 30 µg of protein was loaded onto each lane.

To obtain the rate of fatty acid release by pancreatic lipases, the amount of free fatty acid (FFA) liberated from the CPI-stabilized emulsions was determined by a titration method [27]. Briefly, 5 mL of the sample collected from the small intestine digestion phase was mixed with 10 mL of acetone to inactivate the enzymes, and then titrated with 0.1 M NaOH using phenolphthalein (0.1% (*w*/*v*)) as an indicator. The percentage of FFA was calculated as follows:

(4) $$\mathrm{FFA}\;\left(\%\right)=\frac{V_{NaOH}\times m_{NaOH}\times M_{lipid}}{W_{lipid}\times 2}\times 100$$

where *V_NaOH_* is the titration volume (L), *m_NaOH_* is the molarity of *NaOH* (mol/L), *M_lipid_* is the molecular weight of soybean oil (873 g/mol), and *W_lipid_* is the total weight of soybean oil.

### 2.13. Statistical Analysis

All experiments were repeated 2–3 times as independent replications, and individual tests were performed at least in triplicate. The mean values are reported. Analysis of variance was conducted with SPSS version 21.0 (IBM, New York, NY, USA). Differences between samples and the effects of the treatments were evaluated by Duncan’s multiple range test (*p* < 0.05).

## 3. Results and Discussion

### 3.1. Phenolics Removal and Color Improvement

**Figure 1:**
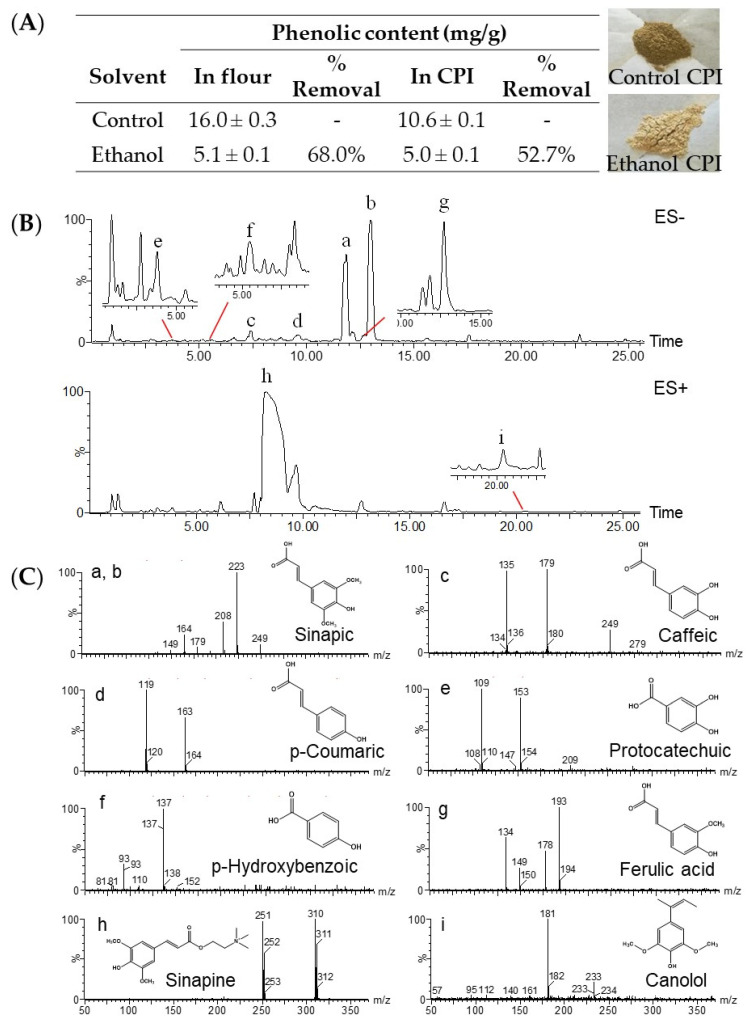
Total phenolic content (mg/g) in canola seed flour and protein isolate (CPI) dephenoled with 70% ethanol (**A**); representative UPLC−TOF−MS chromatogram (**B**); structure (**C**); of phenolic compounds (a–i) extracted from canola flour.

The phenolic content in canola flour and the protein isolate was 16.0 mg/g and 10.6 mg/g, respectively (Figure 1A). The treatment with 70% ethanol extracted and removed most (68.0% and 52.7%) of the endogenous phenolics. Furthermore, the dark greenish color of the control CPI, which was thought to be caused by the alkaline environment applied in protein extraction when phenolic compounds are oxidized to quinones and then bind to proteins [28], changed to light yellow. The intense dark color is one of the main reasons that hinders the acceptability and application of CPI. The removal of polyphenols significantly improved the color characteristic. Of the extracted phenolics identified with LC–MS, sinapic acid was the predominant phenolic acid and sinapine was the most abundant phenolic ester (Figure 1B,C). It has been shown that rapeseed meal contains approximately 1% sinapine, which accounts for the bitter flavor [29]. Minor phenolic acids were p-hydroxybenzoic, protocatechuic, p-coumaric, ferulic, and caffeic acids. Four of the eight phenolic compounds extracted in the present study, caffeic acid, ferulic acid, sinapic acid, and p-coumaric acid, have also been reported [30].

### 3.2. Protein Solubility and Polypeptide Profile

**Figure 2:**
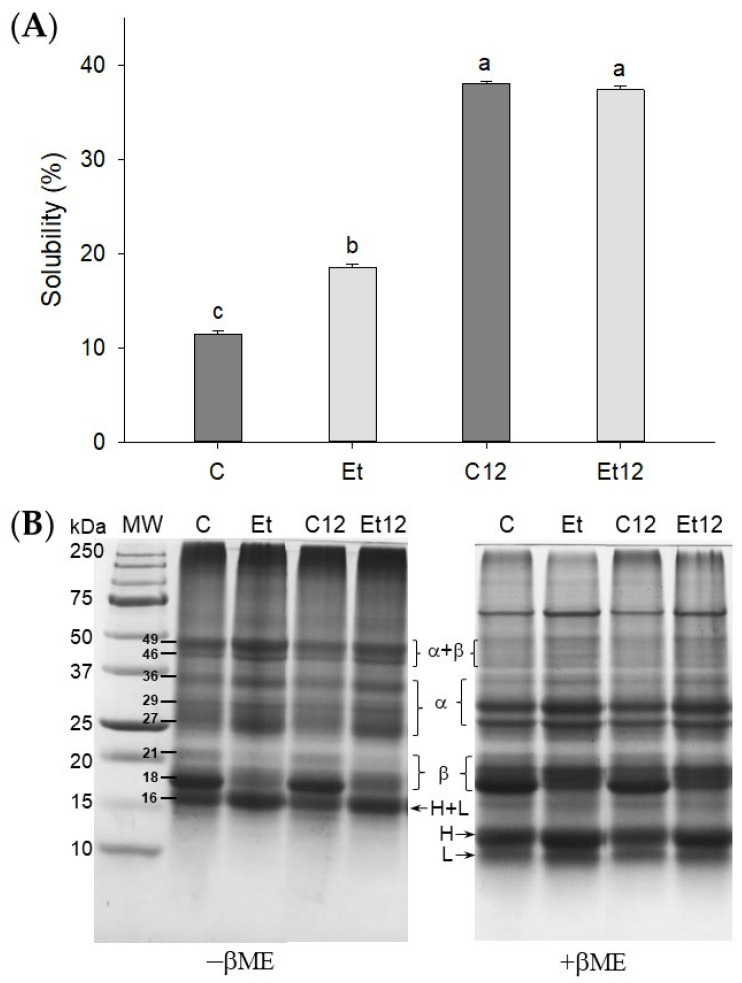
Protein solubility (**A**) and SDS–PAGE (**B**) of dephenoled CPI without or with pH_12_ shift treatment. Samples C: control; Et: dephenoled with 70% ethanol; C12 and Et12: pH_12_ shift treated samples. Bar values with different letters (a–c) are significantly different (*p* < 0.05). βME: β-mercaptoethanol; MW: molecular weight standard; α, β, H, and L: subunits of CPI.

Interactions of endogenous phenolics with polypeptides are a main impediment for solubility and functionality of many plant proteins [31,32], especially for canola protein, which is rich in polyphenols [33]. As shown in Figure 2A, the dephenol extraction treatment significantly improved CPI solubility (58%, *p* < 0.05). The effect can be explained because polyphenols are soluble in organic solvents but are generally insoluble in water; therefore, the removal of bound phenolics will lead to an improved protein–water interaction. Compared with phenol removal, the pH_12_ shift exerted a more pronounced effect on increasing the solubility of CPI. The positive pH_12_ shift effect was seen for both the control and dephenoled samples, although the control CPI remained dark, similar to that shown in Figure 1A. The dissociation of protein subunits is accredited for the improved solubility of legume proteins [14].

To determine whether the solubility change might stem from protein cross-linking, SDS–PAGE was performed under both reducing (+βME) and non-reducing (–βME) conditions. As shown in Figure 2B, eight major bands were identified in non-reducing CPI, which are assigned to cruciferin (18, 21, 27, 29, 36, 44, and 49 kDa) and napin (16 kDa), similar to the report of Wu and Muir [34]. In the presence of βME, the 44 and 49 bands disappeared, suggesting they were disulfide-linked subunits (α–β polypeptides). The 16 kDa polypeptide corresponding to 2S albumin (napin) also faded due to the breakage of the disulfide bond. Correspondingly, new bands attributed to the light chain of 10 kDa (band L) and the heavy chain of 12 kDa (band H) appeared under reducing conditions.

The effect of phenol removal was remarkable. The dephenoled CPI exhibited the fading of β subunit (18, 21 kDa) but an enrichment of polypeptides (16, 27, 49 kDa) (Figure 2B). The dephenol process appeared to increase the exposure of cysteine residues and promoted disulfide bond formation. The 18 kDa band of these samples was recovered under reducing conditions due to the disruption of oleosin protein aggregation from the oil body [4]. Both phenolic compounds and phospholipids, bound to proteins and removed by ethanol, could form a certain steric hindrance to prevent disulfide from bridging between oligopeptides, exemplified by the size increase from 18 Da to 27 Da. Upon the pH_12_ shift, the band intensity for α + β and 16 kDa decreased slightly from their respective control samples. The reason is that the pH_12_ shift facilitates SH/S–S exchange and produces large soluble aggregates that are unable to enter the separating gel [14].

### 3.3. Particle Size and Distribution

**Figure 3:**
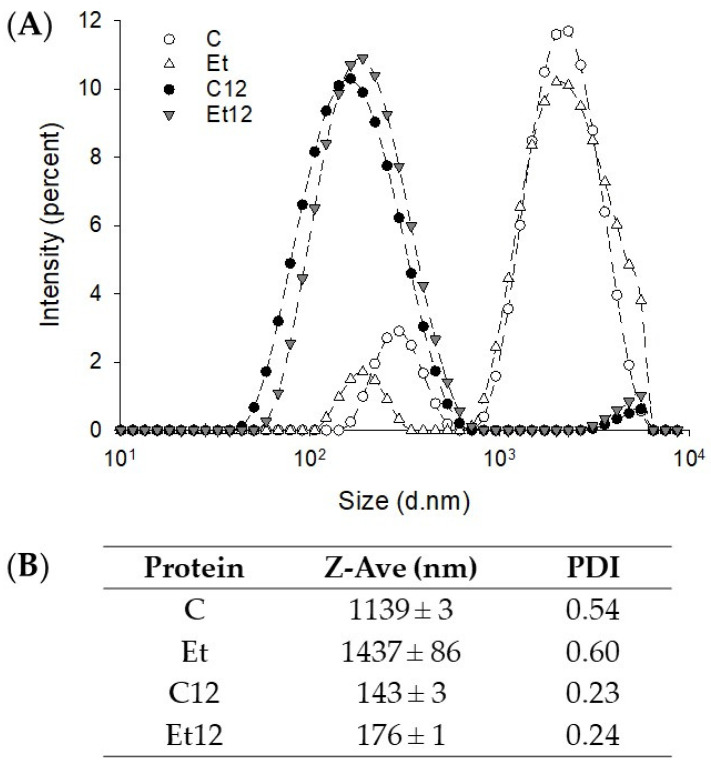
The intensity-diameter distribution (**A**) and Z-average diameter (**B**) of dephenoled canola protein isolate with or without pH_12_ shift treatment. PDI: particle dispersion index. Samples: C: control; Et: dephenoled with 70% ethanol; C12, and Et12: pH_12_ shift-treated samples.

The effect of phenol removal and pH_12_ treatment generated some interesting pattern shifts. Without pH_12_ treatment, CPI particles were distributed in two distinct zones: those within 100–800 nm (minor) and those within 900–8000 nm (abundant) (Figure 3A). The particles in the smaller size group were further reduced after phenol removal, while those in the larger size group became slightly larger, resulting in a small increase in the Z-average (Figure 3B). These large particles in the dephenoled protein were soluble aggregates since the solubility was improved (Figure 2A). The distribution curve shifted heavily to the smaller size region after the pH_12_ treatment, indicating the dissociation of protein subunits, and the dephenoled sample had a slightly elevated Z-average. This explains the phenomenon that even though more disulfide-linked aggregation was observed in the SDS–PAGE profile, the solubility still increased remarkably due to subunit dissociation and charge redistribution.

### 3.4. Structural Characterization

**Figure 4:**
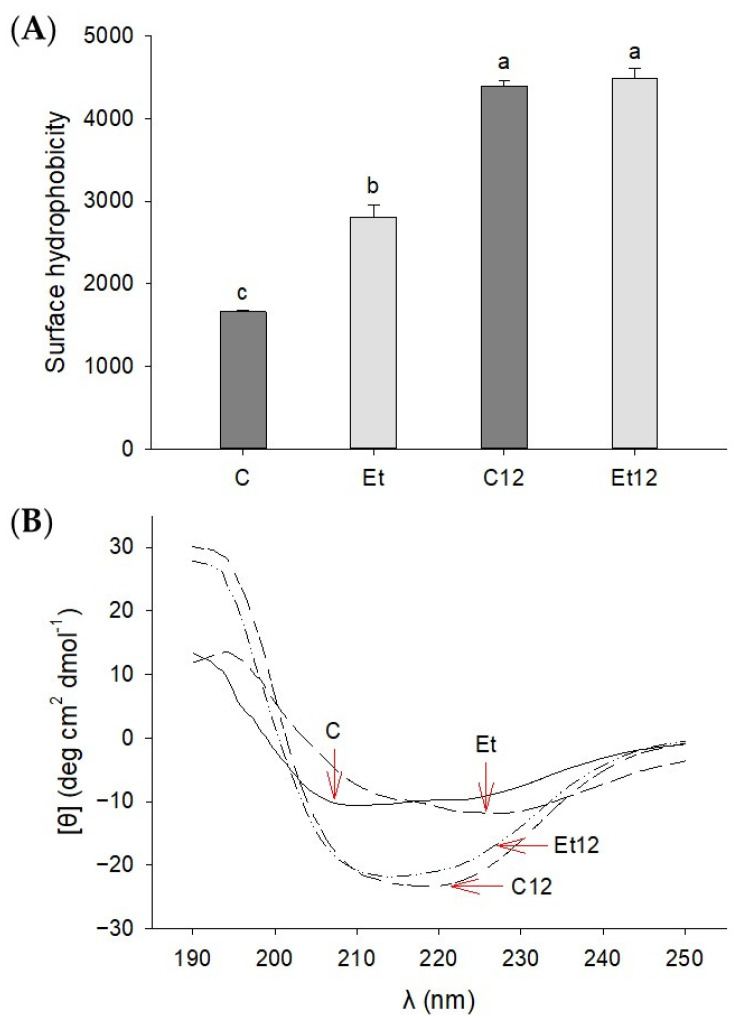
Surface hydrophobicity (**A**) and circular dichroism (**B**) of dephenoled canola protein isolate with or without pH_12_ shift treatment. Samples: C: control; Et: dephenoled with 70% ethanol; C12 and Et12: pH_12_ shift-treated samples. Bar values with different letters (a–c) are significantly different (*p* < 0.05).

In pulses and legumes, many of the globular proteins have a compact structure to shield hydrophobic as well as ionizable groups. The removal of bound polyol polyphenols coupled with pH_12_ shift will disrupt the amphipathic balance to a more hydrophobic state, enabling stronger interactions with the fluorescent probe (ANS) for surface hydrophobicity detection. The results presented in Figure 4A provide strong evidence of such structural changes where the dephenol process alone could increase the hydrophobicity by approximately 63% (*p* < 0.05). A previous study has also reported phenolic removal could decrease hydrophilicity of sunflower protein due to the depletion of hydroxyl and carboxyl groups attributed to phenolic compounds [35]. We have previously shown that soy protein subjected to pH_12_ shift achieved a “molten globule” state, where partially unfolded protein molecules still maintained a relatively intact conformation [14]. Proteins in this state exhibited an increased solubility and hydrophobicity.

The CD spectroscopy also displayed remarkable changes in the secondary structure of the CPI induced by phenol removal and pH_12_ treatment (Figure 4B). In the control CPI, the negative peaks at 222 nm and 208 nm represent the α-helical conformation [36]. After the ethanol phenol extraction, the CD spectra showed a slight red shift. With the pH_12_ treatment, the CD spectra of both control and dephenoled CPI samples showed attenuated ellipticity, suggesting partial disruptions of α-helix. These results indicate that both the phenol removal and pH_12_ shift altered the stability of secondary structures, and the effect of the pH_12_ treatment was far more pronounced.

### 3.5. Interface Properties

**Figure 5:**
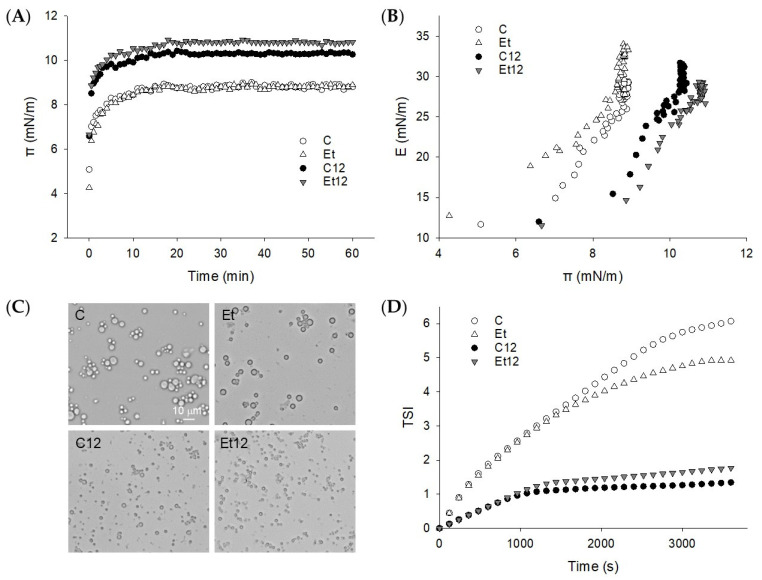
Dynamic surface pressure π-t curve (**A**) and surface dilatational modulus E-π curve (**B**) of proteins adsorbed at the oil–water interface. (**C**): light microscopy of fresh emulsions; (**D**): Turbiscan stable index (TSI) of the emulsions. Samples: C: control; Et: dephenoled with 70% ethanol; C12 and Et12: pH_12_ shift-treated samples.

During the oil–water interface formation, the protein adsorbed rapidly, establishing a strong interfacial pressure (π) within the first 2 min (Figure 5A). Hydrophobic protein–oil interaction is considered to be the primary driving force [37]. Subsequent adsorption and loading were slow and stagnant up to the 60 min testing period. The rapid adsorption suggests that for all the CPI samples, the expansion and rearrangement of the protein molecules at the interface, rather than the diffusion, dominated the event of interfacial tension reduction. The effect of dephenol was complex. While dephenol alone did not alter the pattern of π development (Figure 5A), it significantly increased the dilatational modulus (E) of the interface at an equal π basis (Figure 5B). This suggests that the protein film made by dephenoled CPI was better structured and hence, more elastic. As discussed above, the removal of the steric hindrance of phenolic compounds may promote the interaction between polypeptides to form a cohesive interfacial film. For globular proteins, an increase in E with π reflects the effect of protein interactions on interfacial rheology [38].

The pH_12_ treatment promoted protein adsorption and the initial increase of π was more rapid, especially for dephenoled CPI (Figure 5A). Because protein structure has a profound influence on the diffusion, deployment, and rearrangement during the adsorption process [39], the rate and extent of protein adsorption and loading were obviously enhanced by pH_12_ shift, which modified the protein structure and surface hydrophobicity (Figure 4). Note that at the same surface pressure (π), the E value for dephenoled CPI (Et12) was less than the control (C12). This is attributed to the disulfide bond formation due to the pH_12_ treatment that compromised the viscoelasticity of the protein film at the interface [14].

### 3.6. Emulsion Characterization

The O/W emulsions prepared with the four different CPI samples differed considerably in oil droplet distribution (Figure 5C). The control emulsion had a characteristic of aggregated droplets, and the particle size appeared to be diverse. The degree of such droplet aggregation was slightly reduced when the oil was emulsified with dephenoled protein (Et). The more elastic protein interface built by the dephenoled CPI, as suggested by the dilatational modulus, probably minimized collision-dependent particle association. On the other hand, when prepared with pH_12_ shift-treated protein, the emulsion droplets were nano-scaled, uniform, and well-dispersed. No appreciable difference due to dephenol was seen. Furthermore, dephenol improved the storage stability (less TSI) when the pH_12_ shift was not applied, but this trend was reserved for pH_12_-treated CPI (Figure 5D). This observation is in corroboration with the dilatational modulus test that demonstrated a similar dephenol and pH_12_ shift effects, underscoring the importance of the structure and rheology of interfacial proteins [39]. Moreover, the superior emulsifying activity (particle size reduction) and emulsion stability of the pH_12_ shift-treated CPI, and, to some extent, the dephenoled CPI, can be attributed to the increased protein solubility. The latter has also been reported for other globular proteins [31]. It must be stated that the emulsion stability cannot be fully described in reference to interfacial rheological properties. Inconsistencies and a lack of a close relationship between the two attributes have been noted for milk protein treated with epigallocatechin gallate (EGCG), gallic acid (GA), or tea polyphenols [40]. The removal of endogenous phenols and pH_12_ shift confer complicated protein surface behavior. Therefore, besides the measurement of surface pressure and rheology, other analytical tools may be warranted.

### 3.7. Emulsion Digestion

**Figure 6:**
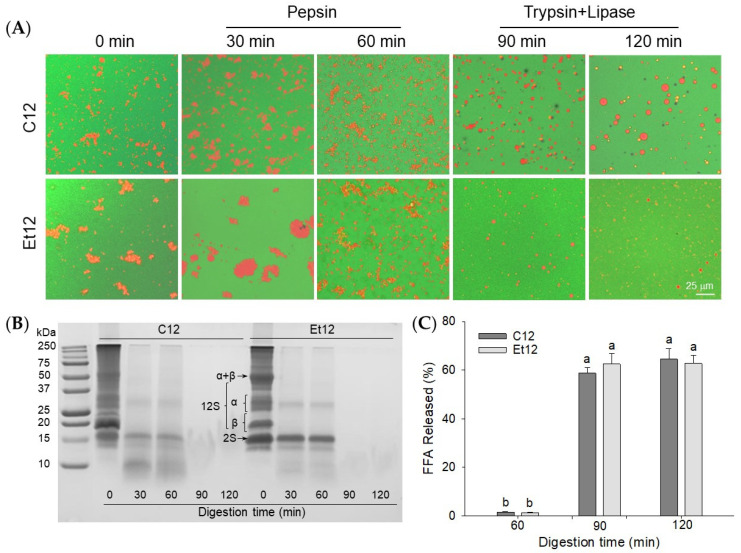
Confocal laser scanning microscopic images (**A**), SDS–PAGE of supernatant proteins (**B**), and percentage of free fatty acids (FFA) released (**C**) for in vitro digested emulsions. The emulsions were prepared with the pH_12_ shift-treated control CPI (C12) or ethanol dephenoled CPI (Et12). α, β, 2S, and 11S denote canola protein subunits. Means with different letters (a,b) differ significantly (*p* < 0.05).

The morphological changes of emulsions during simulated in vitro digestion were examined by confocal microscopy using pH_12_ shift-treated samples as models (Figure 6A). In the acidic gastric solution, flocculation and aggregation of droplets within the Et12 protein emulsion were visibly more pronounced than within the C12 sample. After 30 min of pepsin digestion, extensive aggregation was observed in both the C12 and Et12 emulsions, especially the latter. It can be hypothesized that the less compact protein structure after the ethanol dephenol process facilitated the binding of the membrane protein to the enzyme. The degradation of the interfacial proteins by pepsin led to the destabilization and promoted the aggregation of emulsion droplets [41]. After 60 min of pepsin digestion, the oil droplets became smaller, and their distribution was more uniform. Interestingly, most CPI degradation occurred within the initial 30 min of the digestion, the 12S (α and β) was mostly hydrolyzed, and the 2S (18 kDa) polypeptide was the only remnant component, especially in the Et emulsion digests (Figure 6B). The results support previous observations [42].

After 30 min of subsequent lipase and trypsin digestion (90 min total time), the oil droplets in the C12 emulsion were attenuated yet remained salient. This was in contrast with the Et12 emulsion, where the population of oil droplets was not only small but substantially reduced. This suggests that the dephenol treatment could improve the digestibility of CPI-based O/W emulsions. No protein bands were detected by SDS–PAGE; only very short oligopeptides and free amino acids were present in the intestine digestion stage. As expected, most lipids were hydrolyzed within the 30 min of pancreatic digestion (*p* < 0.05) (Figure 6C). It has been shown that at the intestine digestion stage, the released free fatty acids (and bile salts) competitively displaced proteins at the interface to form a more stable emulsion [41]. This would account for the dispersion of remnant emulsion droplets which were still visible during pancreatic digestion, especially in C12 samples. The high efficacy of pancreatic lipases noted in the present study was in agreement with similar findings reported by [43,44] for protein-stabilized O/W emulsions.

## 4. Conclusions

The dephenol process utilizing 70% ethanol extraction proved to be effective in eliminating structural hindrances of endogenous phenolic compounds to canola seed protein functionality, and it significantly improved the color of the protein isolate. The enhanced conformational flexibility and surface activity thus improved the solubility and emulsifying properties of CPI and were accentuated by the pH_12_ shift treatment, which induced further unfolding and protein particle size reduction. With the combination treatment (dephenol and pH_12_ shift), the resultant CPI as an oil–water interface stabilizer could effectively disperse vegetable oil into nanoparticles. Such emulsions were well digestible under simulated gastrointestinal conditions, exhibiting nearly 100% protein digestibility and >60% fatty acid release. Of particular note for the present investigation, protein-bound endogenous phenolic compounds collectively impeded protein functionality due to their structural diversity and affinity, and this phenomenon was different from many published results that were based on the interaction of individually selected exogenous phenolic compounds with purified proteins. Overall, the combination of ethanol extraction (to remove endogenous phenolics) and pH_12_ shift (to modify protein structure) offers the benefits of improving the aesthetic (color) characteristic as well as the solubility and emulsifying properties of CPI. Hence, it may be adopted as a potential means to overcome the poor functionality constraint of canola seed protein and possibly other seed proteins as well.

## Data Availability

The data presented in this study are as described in the individual figures and tables.
